# Low thiamine status in adults following low-carbohydrate / ketogenic diets: a cross-sectional comparative study of micronutrient intake and status

**DOI:** 10.1007/s00394-024-03459-y

**Published:** 2024-07-05

**Authors:** Chaitong Churuangsuk, Anthony Catchpole, Dinesh Talwar, Paul Welsh, Naveed Sattar, Michael E.J. Lean, Emilie Combet

**Affiliations:** 1 Human Nutrition, School of Medicine, Dentistry, and Nursing, College of Medical, Veterinary and Life Sciences, University of Glasgow, Glasgow Royal Infirmary, Room 2.22, Level 2, New Lister Building 10-16 Alexandra Parade, Glasgow, G31 2ER UK; 2 Department of Internal Medicine, Faculty of Medicine, Prince of Songkla University, Songkhla, Thailand; 3 Scottish Trace Element and Micronutrient Diagnostic and Research Laboratory, Glasgow Royal Infirmary, Glasgow, UK; 4 School of Cardiovascular and Metabolic Health, BHF Glasgow Cardiovascular Research Centre, University of Glasgow, Glasgow, UK

**Keywords:** Ketogenic diet, Low carbohydrate, Magnesium, Thiamine, Micronutrient, Obesity

## Abstract

**Background:**

Low-carbohydrate diets (LCD) are popular for weight loss but lack evidence about micronutrient sufficiency in real-life use. This study assessed the intake and biochemical status of selected micronutrients in people voluntarily following LCDs.

**Methods:**

A cross-sectional study was conducted (2018-20) among 98 adults recruited as self-reporting either LCD (*n* = 49) or diets not restricting carbohydrates (controls; *n* = 49). Diets were assessed using the 130-item EPIC-Norfolk food-frequency questionnaire. Red-blood-cell thiamine diphosphate (TDP) was measured for thiamine status using HPLC. Plasma magnesium, zinc, copper, and selenium were measured using inductively coupled plasma mass spectrometry. Between-group biomarker comparisons were conducted using ANCOVA and adjusted for age, sex, body mass index (BMI), and diabetes status.

**Results:**

LCD-followers (26% male, median age 36 years, median BMI 24.2 kg/m^2^) reported adhering to LCDs for a median duration of 9 months (IQR 4–36). The most followed LCD type was ‘their own variations of LCD’ (30%), followed by ketogenic (23%), ‘palaeolithic’ (15%), and Atkins diets (8%). Among controls, 41% were male (median age 27 years, median BMI 23 kg/m^2^). Median macronutrient intakes for LCD vs control groups were carbohydrate 16%Energy (E) vs. 50%E; protein 25%E vs. 19%E; and fat 55%E vs 34%E (saturated fat 18%E vs. 11%E). Two-thirds of LCD followers (32/49) and half of the controls (24/49) reported some use of dietary supplements (*p* = 0.19). Among LCD-followers, assessing from food data only, 21 (43%) failed to meet the reference nutrient intake (RNI) for thiamine (vs.14% controls, *p* = 0.002). When thiamine from supplementation (single- or multivitamin) was included, there appeared to be no difference in thiamine intake between groups. Still, red-blood-cell TDP was lower in LCD-followers than controls (407 ± 91 vs. 633 ± 234 ng/gHb, *p* < 0.001). Three LCD-followers were thiamine-deficient (RBC thiamine < 275 ng/gHb) vs. one control. There were no significant differences in dietary intakes or plasma concentrations of magnesium, zinc, copper, and selenium between groups.

**Conclusions:**

Following LCDs is associated with lower thiamine intake and TDP status than diets without carbohydrate restriction, incompletely corrected by supplement use. These data, coupled with a lack of RCT evidence on body weight control, do not support recommending LCDs for weight management without appropriate guidance and diet supplementation.

**Supplementary Information:**

The online version contains supplementary material available at 10.1007/s00394-024-03459-y.

## Introduction

Low-carbohydrate and ketogenic diets (LCDs) have become popular for weight and diabetes management [1, 2] and are regularly promoted in the media [3, 4]. Given a lack of difference from higher carbohydrate diets in weight change outcomes, guidelines from Diabetes UK, the European Association for the Study of Diabetes, and the American Diabetes Association suggest that diets with a wide range of macronutrient compositions can be recommended [1, 5, 6]. However, the safety and potential for micronutrient deficiency have been questioned [2, 7].

LCDs have been generally defined as the consumption of carbohydrates less than 26% of total energy intake (or less than 130 g/day), while ketogenic diets consume carbohydrates less than 10% of total energy intake (or less than 20–50 g/day) [8]. LCDs usually restrict fruits, seeds, pulses, and cereal grains and flours used to make bread, pasta and other carbohydrate-rich foods, incurring low intakes of the essential micronutrients contained in these foods. Low intakes of B vitamins, iron, magnesium, and fibre have been reported with LCDs [7, 9, 10], and severe life-threatening thiamine deficiency has been reported with LCD in case studies [11, 12].

Dietary thiamine (B1) is mainly obtained from high carbohydrate foods made with wholegrain or fortified flour and smaller amounts from meat and fish. Magnesium, present in many foods but particularly in high-carbohydrate grains and pulses, is involved in insulin secretion and sensitivity mechanisms; a normal magnesium status protects against type 2 diabetes [13]. A meta-analysis of prospective, population-based, observational studies showed that a 100 mg/day greater dietary magnesium intake (~ 37% of the UK recommendation) was associated with a 20% reduction in the likelihood of having type 2 diabetes, and 10% lower mortality [14, 15].

Outside a small number of clinical trials [7, 9, 10], very little evidence is available about the micronutrient intakes and status of active LCD-followers, despite the media-led popularity, either in healthy adults or in people using the diet to manage their T2D. A cross-sectional study in Iceland reported that half of the LCD followers had thiamine and magnesium intakes below recommended daily intakes [16], raising concerns about potential micronutrient deficiencies with longer-term LCD adherence. In a previous cross-sectional survey from the UK, we reported that only 10% of all LCD followers regularly used multivitamin/mineral supplements [17]. On the other hand, recent evidence shows that any restrictive diets (e.g., LCDs, vegan diets, omnivorous diets) could potentially lead to inadequate micronutrient intakes, but the evidence on body status of micronutrients is still limited [7, 9, 10, 18].

The present study aimed to establish dietary habits, consumptions of macro- and micronutrients, and body status of micronutrients, particularly thiamine and magnesium, of people voluntarily following LCDs, compared to a sample of individuals who had not modified their diets. Zinc, copper, and selenium were also of interest, as little evidence has been reported.

## Materials and methods

### Study design and study population

A cross-sectional comparative study was conducted to document dietary intake, micronutrient status (specifically thiamine and magnesium status) and body composition in adults following LCDs, compared with people whose diets had normal carbohydrate intake (control group). The study was approved by the University of Glasgow Research Ethics Committee (Project No. 200,170,032).

Participants were recruited using local advertisements (posters and leaflets in public places and on social media) between February 2018 and January 2020, seeking volunteers aged 18 and above, (i) who had followed LCDs for at least 1 month, and (ii) others (controls) who were not eating any specific diet. Exclusion criteria were pregnancy and lactation; having gastrointestinal tract disease that may affect diet or nutrient absorption, such as coeliac disease or inflammatory bowel disease; currently participating in other intervention studies. Written informed consent was obtained from all participants.

### Outcome measures

Outcomes of the present study included dietary intake, anthropometric data, body composition, thiamine status determined by red blood cell thiamine diphosphate (RBC-TDP), and magnesium status determined by plasma magnesium. Plasma copper, zinc and selenium were also analysed.

We set out primarily to analyse and present the results according to how participants defined themselves as LCD followers or as people who did not restrict dietary carbohydrates. We recognised that their actual dietary choices around carbohydrate-rich foods could influence other dietary behaviours, affecting micronutrient intake and status. Therefore, measuring markers of micronutrient status is also important.

### Dietary intake assessment

Dietary intake was assessed with the EPIC-Norfolk food frequency questionnaire (FFQ) to capture habitual intake over one year (or up to the start of LCDs) and was analysed using the FETA software [19]. The questionnaire consists of 130 food items with portion size attached to each item and nine frequency categories from ‘never or less than once per month’ to ‘6 + times per day’. Participants filled out the FFQ by themselves after an explanation by the researcher. Entries were checked for completeness (< 10 lines missing). A single, multi-pass 24-h recall was also collected as a backup in case of missing/incomplete FFQ data: 24-h recall data were used for four participants (one with incomplete FFQ [missing ticks for more than 10 lines], two who did not return the FFQ questionnaires, and one with implausible energy intake of 12,472 kcal/day).

To collect information on dietary supplementation, we asked participants for the brands and the dose of supplements they took. If they could not remember, we helped them search on the internet or took a photo of the packaging for us to find out later. Knowing the brand and dose, we could track down the amount of micronutrients from product information.

### Dietary behaviour of LCD-followers

LCD followers filled out additional questionnaires about their dietary habits (type of LCDs followed, duration of LCDs, source of information for LCDs, motivation to follow LCDs, portion size control during LCDs), and engagement with health practitioners.

### Blood collection

Participants fasted overnight for at least 8 h before the morning appointment, and venous blood was collected for thiamine, magnesium, zinc, copper, and selenium status measurements. Blood samples were centrifuged at 3500 rpm for 15 min at 4 °C. Plasma was removed, packed red cells were prepared by carefully removing all remaining plasma and buffy coat, and stored at -20 °C.

### Micronutrient analysis

Micronutrients were assayed at the Scottish Trace Element and Micronutrient Diagnostic and Research Laboratory, a national accredited service. Thiamine as TDP was measured in RBCs using HPLC with post-column ferricyanide derivatisation and fluorometric detection [20]. The TDP concentration was related to haemoglobin (Hb) in the sample (ng TDP/g Hb), and the value below 275 ng/g Hb was considered thiamine deficiency. Lithium heparin plasma was used to measure magnesium, copper, zinc, and selenium using inductively coupled plasma mass spectrometry (ICP-MS, Agilent Technologies, Cheadle, UK) as previously published [21]. Reference ranges for thiamine, plasma copper, zinc, and selenium were obtained from the Scottish Trace Element and Micronutrient Diagnostic and Research Laboratory. For plasma magnesium, the ICP-MS method was mainly used for research; thus, the reference range was obtained from the literature, and plasma magnesium below 0.75 mmol/l was considered hypomagnesaemia [22, 23].

### Statistical analysis

The primary aim of the present study is to compare thiamine and magnesium intakes and status in self-reported followers of LCD and non-restricted diets. Our recent systematic review found no reports of thiamine and magnesium status in individuals following LCDs despite the metabolic importance of these two micronutrients [7]. Therefore, sample size calculation was based on intakes of thiamine and magnesium being reduced on LCDs, as reported in an RCT by Gardner et al. [24]. Compared to a higher carbohydrate diet for 8 weeks, the Atkins diet caused lower intakes of thiamine (0.9 ± 0.4 vs. 1.4 ± 0.4 mg/day) and magnesium (231 ± 86 vs. 286 ± 89 mg/day). With an alpha of 5% and 80% power to detect differences between the two diets, 82 participants were needed (41 participants per group).

Because nutrient intakes were not normally distributed, non-parametric tests were used to examine differences between groups in macro- and micronutrient intakes. Dietary intakes were also compared to UK Dietary Reference Value, which includes Reference Nutrient Intake (RNI, a level of intake considered sufficient to meet the requirements of 97.5% of the population group) and Lower Reference Nutrient Intake (LRNI, a level of intake that is enough for only a small number (2.5%) of population group but this level is not enough for most people) [25].

For biomarkers, differences in RBC-TDP (thiamine), plasma magnesium, copper, zinc and selenium between groups were analysed using ANCOVA, adjusted for sex, age, BMI, and diabetes status, due to an imbalance of such population characteristics between groups.

A *post hoc* analysis was also conducted based on group re-allocation that was aligned with practice (actual intake of LCDs) rather than self-identified. All participants were re-grouped using the cut-off carbohydrate intake < 130 g/day (a widely accepted definition of LCD [26, 27]), as the LCD group, namely ‘reallocated true LCD’, and ≥ 130 g/day as the normal diet group. Differences in dietary intake, thiamine, magnesium, zinc, copper, and selenium status between the two new groups were also compared.

Subgroup analyses for thiamine status in participants with and without thiamine supplement (either as a single supplement or combined with other nutrients such as multivitamins) and magnesium status with and without magnesium supplement (either as a single supplement or combined with other nutrients such as multivitamins) were also conducted. *P*-value < 0.05 was considered statistically significant. Analyses were conducted in SPSS and R programs with packages ‘epicalc’ and ‘ggplot2’.

## Results

### Participant characteristics

A total of 98 adults (49 self-reported LCD followers vs. 49 controls) participated in the study (Table 1). Most participants (65/98) were women. LCD-followers had a higher median age (36, IQR 25–49) than controls (27, IQR 24–34, *p* < 0.001). Median BMI was similar in the two groups (LCD 24.2 kg/m^2^, control 23.1 kg/m^2^). 10% of the LCD group had type 2 diabetes (*n* = 5/49), while no diabetes was reported in the control group. Two-thirds of LCD-followers and half of the controls reported using dietary supplements, with no evidence for difference between groups. When participants were reallocated according to their carbohydrate intake, 7 in the LCD and 6 in the control groups were misclassified.

**Table 1:** Characteristics of the participants (*n* = 98)

Characteristics	LCD-followers *n* = 49	Controls *n* = 49	*P*-value
**Women, n (%)**	36 (74)	29 (59)	0.200 ^a^
**Age (years), median (IQR)**	36 (25, 49)	27 (24, 34)	***< 0.001*** ^b^
**Body mass index (kg/m** ^**2**^ **), median (IQR)**	24.2 (21.2, 28.7)	23.1 (20.3, 25.8)	0.063 ^b^
**Body mass index, n (%)**			0.151 ^c^
- Underweight	1 (2)	3 (6)	
- Normal weight	26 (53)	33 (66)	
- Overweight	14 (29)	12 (24)	
- Obesity	8 (16)	2 (4)	
**Waist circumference (cm), median (IQR)**	84 (74, 93)	80 (73, 89)	0.251 ^b^
**Pulse rate (bpm), mean (SD)** ^e^	68 (12)	70 (10)	0.250 ^d^
**Ethnicity, n (%)**			***0.007*** ^c^
- White	39 (80)	25 (51)	
- Asian	6 (12)	18 (37)	
- Other	4 (8)	6 (12)	
**Education, n (%)**			***0.001*** ^c^
- School leaver/standard grade/GCSE	2 (4)	1 (2)	
- Higher/A-level	2 (4)	5 (10)	
- Higher education HND/HNC/NVQ	5 (10)	0	
- Bachelor	20 (41)	8 (16)	
- MSc/PhD/Postgraduates	20 (41)	35 (71)	
**Supplement use, n (%)**			
- any supplement	32 (65)	24 (50)	0.187 ^a^
- combined multivitamin or B vitamin	16 (33)	12 (24)	0.371 ^a^
- multivitamins	11 (22)	7 (14)	0.297 ^a^
- multi-minerals	2 (4)	3 (6)	1.000 ^c^
- vitamin B (any)	7 (14)	5 (10)	0.538 ^a^
- vitamin C	5 (10)	7 (14)	0.538 ^a^
- vitamin D	14 (29	8 (16)	0.268 ^a^
- magnesium	6 (12)	2 (4)	0.268 ^c^
- calcium	3 (6)	1 (2)	0.617 ^c^
- iron	1 (2)	1 (2)	1.000 ^c^
**Comorbid diseases, n (%)**	15 (31)	6 (13)	***0.027*** ^a^
- Type 2 Diabetes	5 (10)	0	0.056 ^c^
- Type 1 Diabetes	2 (4)	0	0.495 ^c^
- Hypertension	4 (8)	1 (2)	0.362 ^c^
**Proportion who met the definition of LCD**			
***as grams of CHO***, **n (%)**			
True LCD (< 130 g)	42 (86)	6 (12)	-
*- Ketogenic (< 50 g)*	*22 (45)*	*0*	-
*- LCD (50 to < 130 g)*	*20 (41)*	*6 (12)*	-
Normal CHO (≥ 130 g)	7 (14)	43 (88)	-
***as % energy intake***, **n (%)**			
True LCD (< 26%E)	33 (68)	0	-
- Ketogenic (< 10%E)	12 (25)	0	-
- LCD (10 to < 26%E)	21 (43)	0	-
Normal CHO (≥ 26%E)	16 (33)	49 (100)	-

Data are proportion and percentage [n (%)], unless otherwise indicated

*P*-value < 0.05 was considered statistically significant

IQR, interquartile range; LCD, low-carbohydrate diet; SBP, systolic blood pressure; DBP, diastolic blood pressure; CVD, cardiovascular diseases; CHO, carbohydrate; E, energy intake; ns, not significant

^a^ Chi-square tests

^b^ Rank sum tests

^c^ Fisher’s exact tests

^d^ Independent samples T-tests

^e^ Participants with hypertension were excluded

### Dietary behaviour of LCD-followers

Of 49 LCD-followers, 42 reported the type of diet they followed, their motivation and source of information. The most often followed LCD type was personal ‘variations of a LCD’ (30%), followed by ketogenic diets (23%) and palaeolithic diets (15%). The Atkins diet only accounted for 8%. The median duration for adherence to LCDs was 9 months (IQR 4–36). Reported mean weight loss was 12 kg since commencing the LCD. The top-ranked reason to follow LCDs was ‘better for health’, which accounted for 45% of LCD-followers, followed by ‘weight loss’ (24%) and the belief of being ‘allergic to gluten’ (12%). Only 2 participants followed LCDs for diabetes management. Portion size varied, with 45% reporting smaller portion sizes, 31% larger, and 24% reporting no change. One-fifth of the LCD followers reported that their GP knew they were following LCDs (21%). Additionally, only 14% of LCD followers had met a dietitian or nurse for dietary advice. On the other hand, information from the internet (e.g., blogs, forums) was the most trusted source of dietary advice (43%), followed by diet books (17%) and family members (10%).

### Intake of food groups

Intakes of major food groups, estimated from the EPIC-Norfolk FFQ, are presented in Table 2. Intake of cereals and cereal products, potatoes, fruits, sugar preserves and snacks were 2 to 15 times lower in the LCD group than the control group. LCD-followers consumed more (1.5-2 times higher) vegetables and non-alcoholic beverages than controls. Differences in meat, fish and fat intakes were not evident in the present study. In a post-hoc analysis of ‘reallocated true’ LCD vs. normal diet groups, the differences in all food groups mentioned above remained, except for vegetable intake, now similar between groups at approximately 3 to 4 portions per day (Supplementary Table S1).

**Table 2:** Consumption of food groups in self-reported LCD-followers and controls (*n* = 94)

Food groups as grams/day	LCD-followers *n* = 45	Controls *n* = 49	*P*-value ^a^
Cereals and cereal products	18 (0,117)	277 (195,450)	***< 0.001***
Potatoes	9 (0,18)	26 (18,63)	***< 0.001***
Meat and meat products	131 (99,187)	101 (58,173)	0.173
Fish and fish products	48 (24,83)	31 (24,45)	0.094
Eggs and egg dishes.	40 (22,125)	40 (18,40)	0.307
Fats and oils	6 (4,10)	7 (4,11)	0.850
Nuts and seeds	15 (2,28)	8 (2,21)	0.116
Vegetables	337 (245,458)	223 (144,377)	***0.004***
Fruits	90 (47, 215)	182 (96,284)	***0.002***
Soups and sauces	39 (24,65)	50 (28,125)	0.235
Milk and milk products	78 (34,132)	65 (31,160)	0.868
Non-alcoholic beverages	731 (380,950)	345 (206,609)	***0.001***
Sugar preserves and snacks	6 (1,12)	23 (13,46)	***< 0.001***

Data are median and inter-quartile range generated from the FFQ (*n* = 94). Data were missing for 4 participants in the LCD group because 3 FFQs were incomplete, and 1 FFQ was excluded as unreliable because it contained an implausible energy intake of 12,472 kcal/day

*P*-value < 0.05 was considered statistically significant

^a^*P*-value of Mann-Whitney U test

### Energy and macronutrient intake

Median energy intake was lower for LCD-followers (1,291 kcal/day) compared to controls (1,726 kcal/day) (Table 3). The median carbohydrate intake in the LCD group was 53 g/day (16%E), 4 times lower than the control group (206 g/day; 50%E). The LCD group consumed more total fat (55 vs 34%E), saturated fat (18 vs 11%E), mono-unsaturated fat (21 vs 13%E), and poly-unsaturated fat (8 vs 6%E) than the control group. LCD and control groups had similar fibre intake (16 vs. 18 g/day; p = 0.10). Following group re-allocation in a posthoc analysis, median fibre intake was lower (12.5 g/day) in the ‘reallocated true’ LCD group than in the control group (20 g/day; *p* < 0.001; Supplementary Table S2).

**Table 3:** Macronutrient contribution from diet only in self-reported LCD-followers and controls (*n* = 98)

Nutrients	Median of intakes per day			Proportion meeting recommendation, *n* (%)			Recommendations
	LCD-followers *n* = 49	Controls *n* = 49	*P*-value ^a^	LCD-followers *n* = 49	Controls *n* = 49	*P*-value	
Energy (kcal)	1291 (1094, 1630)	1726 (1272, 2088)	***0.003***	-	-	-	-
CHO (%E)	16.0 (10.4,29.4)	49.6 (42.7,53.1)	***< 0.001***	2 (4)	23 (47)	< 0.001 ^c^	50%E
Protein (%E)	24.8 (21.4,28.5)	18.7 (16.4,21.9)	***< 0.001***	-	-	-	-
Fat (%E)	55.3 (45.8,63.1)	33.5 (30.8,39.4)	***< 0.001***	4 (8)	31 (63)	< 0.001 ^c^	< 35%E
SFA (%E)	18.3 (12.9,25.5)	11.3 (9.4,13.5)	***< 0.001***	7 (14)	20 (41)	0.003 ^d^	< 11%E
MUFA (%E)	21.3 (18.1, 23.2)	12.8 (11.7, 15.8)	***< 0.001***	47 (96)	21 (43)	< 0.001 ^d^	13%
PUFA (%E)	8.3 (7.3, 10.3)	6.2 (5.5, 7.2)	***< 0.001***	42 (86)	21 (43)	< 0.001 ^d^	6.5%
Fibre (g) ^b^	16.0 (9.2, 22.0)	18.2 (13.8, 24.3)	0.099	6 (12)	6 (12)	1.000 ^d^	30 g/day
CHO (g)	53.2 (28.5, 110)	206 (147, 259)	***< 0.001***	-	-	-	-
Protein (g)	86.4 (61.1, 100)	81.0 (65.9, 107)	0.946	-	-	-	-
Fat (g)	79.6 (59.3, 93.5)	62.6 (47.9, 86.4)	***0.027***	-	-	-	-
SFA (g)	27.1 (17.4, 38.7)	20.9 (15.5, 29.3)	***0.040***	-	-	-	-
MUFA (g)	30.5 (25.2, 35.2)	24.4 (18.0, 35.8)	***0.019***	-	-	-	-
PUFA (g)	13.0 (9.8, 15.6)	12.2 (10.0, 15.2)	0.488	-	-	-	-

Data are median and interquartile range

*P*-value < 0.05 was considered statistically significant

CHO, carbohydrate; E, energy; SFA, saturated fatty acids; MUFA, mono-unsaturated fatty acids; PUFA, poly-unsaturated fatty acids

^a^ Mann-Whitney U test

^b^ Fibre is presented as AOAC method by multiplying non-starch polysaccharides (Englyst methods obtained from the FFQ) by 1.33 [28]

^c^ Fisher’s exact tests

^d^ Chi-square tests

### Vitamins and minerals intake

The median intakes of vitamins A, B2, B3, B6, B12, folate, and C were higher than the RNI in both groups. When comparing groups, the median thiamine intake in the LCD group was lower than in the control group, whereas vitamins A, D and B12 were higher in the LCD group than in the control group (Table 4). The differences in mineral intake were not evident between groups. However, in reallocated groups (post-hoc analysis), calcium, magnesium, iron, copper, zinc, and selenium intakes were lower in the ‘true’ LCD group than in the control group (Supplementary Table S3).

**Table 4:** Vitamins and minerals from diet only in self-reported LCD-followers and controls (*n* = 98)

Vitamins	LCD-followers *n* = 49	Controls *n* = 49	*P*-value ^a^
A (µg) ^b^	1486 (927, 1870)	934 (638, 1318)	***0.001***
D (mg)	3.7 (1.9, 5.7)	2.6 (1.6, 3.7)	***0.043***
E (mg)	9.7 (7.4, 12.5)	10.4 (8.1, 13.1)	0.597
B1 (mg)	0.97 (0.69, 1.41)	1.34 (1.13, 1.64)	***0.008***
B2 (mg)	1.4 (1.0, 1.9)	1.4 (1.0, 1.7)	0.384
B3 (mg)	21.9 (16.2, 25.9)	22.5 (16.8, 27.5)	0.652
B6 (mg)	1.6 (1.3, 2.1)	2.0 (1.4, 2.4)	0.190
Folate (µg)	267 (214, 351)	262 (192, 308)	0.269
B12 (µg)	8.4 (3.8, 13.6)	5.3 (3.2, 8.3)	***0.028***
C (mg)	108 (62.7, 132)	100 (54.2, 143)	0.688
**Minerals**			
Sodium (mg)	2156 (1425, 2522)	2189 (1667, 2735)	0.428
Potassium (mg)	2656 (1974, 3220)	2970 (2357, 3605)	0.171
Calcium (mg)	596 (422, 726)	619 (502, 815)	0.218
Magnesium (mg)	242 (179, 327)	283 (231, 346)	0.063
Iron (mg)	9.2 (7.1,12.1)	10.4 (8.6, 11.6)	0.420
Copper (mg)	1.1 (0.8, 1.5)	1.3 (0.9, 1.7)	0.069
Zinc (mg)	8.4 (7.0, 10.7)	9.1 (7.4, 11.8)	0.475
Selenium (µg)	57.6 (38.7, 80.6)	66.7 (47.3, 90.0)	0.223
Iodine (µg)	114 (68.5, 171)	121 (84.3, 159)	0.774

Data are in the median and interquartile range, obtained from food records, not including dietary supplements

*P*-value < 0.05 was considered statistically significant

^a^ Mann-Whitney U test

^b^ Vitamin A as retinol equivalent

Table 5 shows the proportion of participants whose dietary intakes met the UK RNI for thiamine, magnesium, calcium, iron, iodine, and selenium because the median intakes of these micronutrients were consumed below the RNI level, indicating inadequate consumption (Supplementary Table S4). Just above half of LCD-followers (57%) met the RNI for thiamine, which was 1.5 times lower than the proportion of controls (86%; *p* = 0.002). Likewise, only 16% of LCD-followers met the RNI for iron, compared to 39% in the control group. Approximately one-third of LCD followers met the RNI for calcium, magnesium, and selenium, compared to 41–55% in the control group. Iodine was the only micronutrient that more LCD-followers (41%) met the RNI than controls (33%). With supplementation, the proportion of LCD followers achieving RNI increased, in varying degrees, to a level where there was no difference between groups for all these micronutrients except for iodine.

**Table 5:** Proportion of participants (*n* = 98) who met UK Reference Nutrient Intake and who were below the UK Lower Reference Nutrient Intake for selected micronutrients as of diet only and including supplementation

	Diet only				With supplementation		
	LCD (*n* = 49)	Controls (*n* = 49)	*p*-value		LCD (*n* = 49)	Controls (*n* = 49)	*p*-value
***N*** **(%) who met RNI**							
Thiamine	28 (57)	42 (86)	***0.002***		34 (69)	42 (86)	0.053
D							
Magnesium	19 (39)	27 (55)	0.105		28 (57)	28 (57)	1.000
Calcium	16 (33)	20 (41)	0.402		17 (35)	21 (43)	0.407
Iron	8 (16)	19 (39)	***0.013***		14 (29)	20 (41)	0.203
Iodine	20 (41)	16 (33)	0.402		20 (41)	18 (37)	0.678
Selenium	18 (37)	23 (47)	0.306		19 (39)	25 (51)	0.223
**N (%) who were below LRNI**							
Thiamine	0	0	na		0	0	na
Magnesium	12 (25)	5 (10)	0.062		8 (16)	5 (10)	0.372
Calcium	8 (16)	5 (10)	0.372		8 (16)	5 (10)	0.372
Iron	11 (22)	11 (22)	1.000		9 (18)	11 (22)	0.616
Iodine	13 (27)	9 (18)	0.333		12 (25)	8 (16)	0.316
Selenium	14 (29)	9 (18)	0.233		14 (29)	8 (16)	0.146

*P*-value obtained from Chi-square test

*P*-value < 0.05 was considered statistically significant

LCD, low-carbohydrate diet; RNI, Reference Nutrient Intake; LRNI, Lower Reference Nutrient Intake

It is of concern that 22–29% of LCD followers had magnesium, iron, iodine, and selenium below the lower reference nutrient intake (LRNI; Table 5). Including intake from supplementation, the proportion of LCD-followers who were below the LRNI improved by 9% for magnesium, while there was a small or no change for other micronutrients. None of the thiamine intakes were below the LRNI value.

### Biomarkers of thiamine, magnesium, zinc, copper, and selenium

**Fig. 1:**
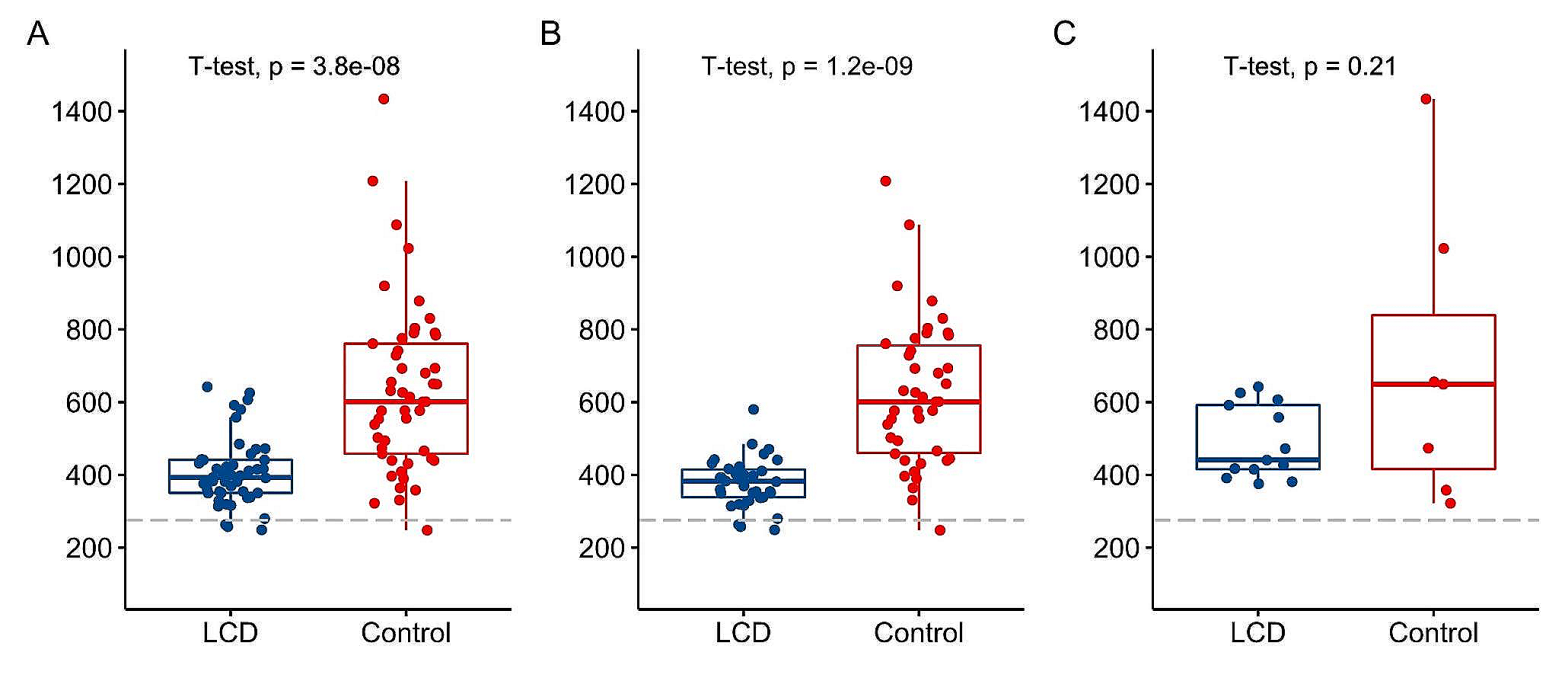
Blood concentration of red blood cell thiamine diphosphate (ng/g Hb) in **(A)** all self-reported LCD-followers (*n* = 49) and controls (*n* = 49); **(B)** in not taking thiamine or multivitamin supplements: 36 LCD-followers vs. 42 controls; **(C)** in those taking supplements: 13 LCD-followers vs. 7 controls **(C).***P*-value ANCOVA tests (adjusted for age, sex, BMI, diabetes status) were < 0.001 for A and B, not significant for C. *P*-value < 0.05 was considered statistically significant

**Fig. 2:**
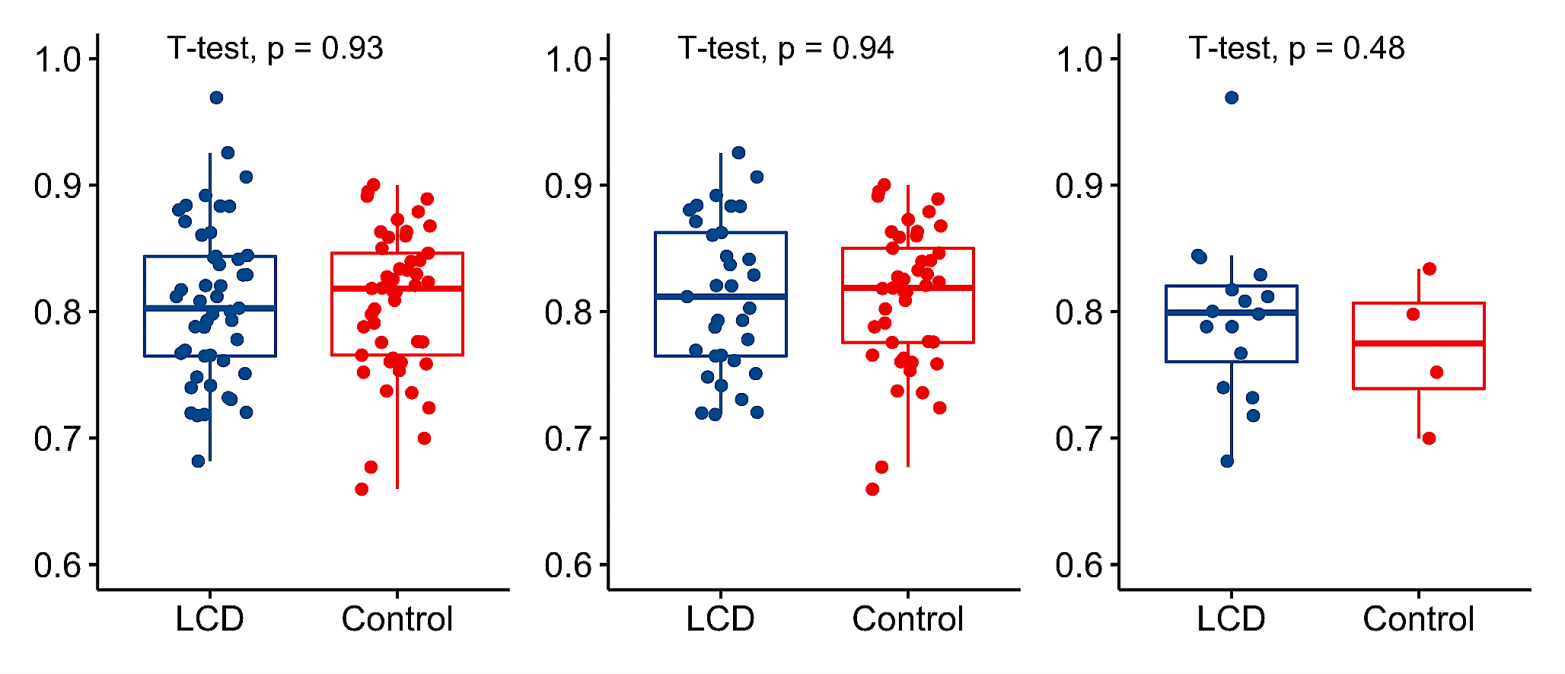
Plasma concentration of magnesium (mmol/l) in **A)** all self-reported LCD-followers (*n* = 49) and controls (*n* = 49); **B)** in not taking magnesium or multimineral supplements: 33 LCD-followers vs. 45 controls; **C**) in those taking supplements: 16 LCD-followers vs. 4 controls **(C).***P*-value ANCOVA tests (adjusted for age, sex, BMI, diabetes status) were not significant for all comparisons. *P*-value < 0.05 was considered statistically significant

Thiamine concentration (as TDP in RBCs expressed in ng per g of Hb) was lower in the LCD group than in the control group (407 ± 91 vs. 633 ± 234 ng/gHb, *p* < 0.001; Table 6; Fig. 1A). Three LCD-followers (6%) had biochemical thiamine deficiency (< 275 ng/gHb), compared to one participant (2%) in the control group. None of them used a thiamine supplement. In a subgroup analysis of participants who did not use thiamine or multivitamin supplements (*n* = 78), LCD-followers had lower TDP concentration than controls (Figs. 2B and 378 ± 68 vs. 621 ± 200 ng/g Hb, *p* < 0.001 & Table 6).

**Table 6:** Blood concentration and status of thiamine, magnesium, zinc, copper and selenium in self-reported LCD-followers and controls (*n* = 98)

	All				Without supplements				With supplements		
	LCD-followers	Controls	*P*-value		LCD	Control	*P*-value		LCD	Control	*P*-value
**Thiamine**	*n* = 49	*n* = 49			*N* = 36	*N* = 42			*N* = 13	*N* = 7	
RBC TDP (ng/g Hb)	407 ± 91	633 ± 234	***< 0.001*** ^**a**^		378 ± 68	621 ± 200	***< 0.001*** ^**a**^		488 ± 101	702 ± 400	0.210 ^a^
Thiamine deficiency, n (%)	3 (6)	1 (2)	0.617 ^b^		3 (8)	1 (2)	0.330 ^b^		0	0	NA
**Magnesium**	*n* = 49	*n* = 49			*N* = 33	*N* = 45			*N* = 16	*N* = 4	
Plasma magnesium (mmol/l)	0.81 ± 0.06	0.81 ± 0.06	0.934 ^a^		0.81 ± 0.06	0.81 ± 0.06	0.940 ^a^		0.80 ± 0.07	0.77 ± 0.06	0.493 ^a^
Hypomagnesaemia, n (%)	10 (20)	6 (12)	0.274 ^c^		6 (18)	5 (11)	0.375 ^c^		4 (25)	1 (25)	1.000 ^b^
**Zinc**	*n* = 49	*n* = 49			*N* = 41	*N* = 45			*N* = 8	*N* = 4	
Plasma zinc (µmol/l)	12.89 ± 1.86	12.48 ± 1.51	0.223 ^a^		12.86 ± 1.94	12.45 ± 1.56	0.280 ^a^		13.06 ± 1.50	12.73 ± 0.91	0.695 ^a^
Hypozincaemia	2 (4)	3 (6)	1.000 ^b^		2 (5)	3 (7)	1.000 ^b^		0	0	NA
**Copper**	*n* = 49	*n* = 49			*N* = 47	*N* = 46			*N* = 2	*N* = 3	
Plasma copper (µmol/l)	16.32 ± 3.50	15.58 ± 4.80	0.876 ^a^		16.41 ± 3.55	15.51 ± 4.93	0.314 ^a^		14.32 ± 0.56	16.67 ± 2.03	0.224 ^a^
Copper deficiency	0	2 (4)	0.495 ^b^		0	2 (4)	0.242 ^b^		0	0	NA
**Selenium**	*n* = 49	*n* = 49			*N* = 47	*N* = 44			*N* = 2	*N* = 5	
Plasma selenium (µmol/l)	1.29 ± 0.16)	1.27 ± 0.21	0.576 ^a^		1.28 ± 0.16	1.23 ± 0.15	0.147 ^a^		1.48 ± 0.03	1.58 ± 0.40	0.607 ^a^
Selenium deficiency	0	0	NA		0	0	NA		0	0	NA

Data are mean ± SD unless otherwise indicated

LCD, low-carbohydrate diet; RBC, red blood cells; TDP, thiamine diphosphate; NA, not applicable

The reference interval for thiamine is 275–675 ng/g Hb; for plasma Mg is 0.75-1.0 mmol/l; for plasma zinc is 11–18 µmol/l in men and 10–18 µmol/l in women; for plasma copper is 10–22 µmol/l in men and 11–25 µmol/l in women; for plasma selenium is 0.75–1.50 µmol/l

^a^*P*-value from independent T test

^b^*P*-value from Fisher’s exact test

^c^*P*-value from Chi-square test

Additionally, TDP concentration showed a good correlation with carbohydrate intake, either as a percentage of energy intake (rho = 0.60, *p* < 0.001) or grams per day (rho = 0.57, *p* < 0.001). Considering the duration of LCD practice and thiamine status, no correlation was found between the duration of LCD practice and TDP concentration (rho= -0.02, *p* = 0.92; *n* = 30).

The difference in plasma magnesium was not evident (Fig. 2; Table 6). However, magnesium deficiency (known as hypomagnesemia – plasma magnesium < 0.75 mmol/l) was more prevalent, accounting for 20% (*n* = 10/49) in the LCD group, nearly double that of the control group (12%, *p* = 0.274; Table 6). Of the LCD-followers who did not use magnesium or multimineral supplements (*n* = 78), 18% (6/33) had hypomagnesemia, compared to 11% (5/45) in controls (*p* = 0.375; Table 6).

There was no statistically significant difference between groups when comparing the proportions with hypomagnesemia between supplementers vs. non-supplementers, regardless of carbohydrate intake. Hypomagnesemia in non-supplementers was *n* = 11/78 (14%) vs. *n* = 5/20 (25%) in supplementers, Chi-square test *p*-value = 0.239.

Differences in plasma zinc, copper, and selenium were also not evident (Table 6).

A posthoc analysis of RBC TDP, plasma magnesium, zinc, copper, and selenium in ‘reallocated true’ LCD vs. control groups showed similar results to the main findings: RBC TDP was lower in the LCD group than control while there were no differences in plasma magnesium, zinc, copper, and selenium between groups (Supplementary Table S5).

## Discussion

Evidence is lacking regarding micronutrient status in people who voluntarily follow LCDs in real-life settings. The present study shows, for the first time, that LCD-followers have lower blood thiamine levels (with 6% [*n* = 3/49] in a deficient state) than individuals with higher carbohydrate intake. We also found that hypomagnesemia (plasma magnesium < 0.75 mmol/l) was more prevalent in LCD-followers, approximately 20% compared to 12% in the normal diet group. This mirrors sub-optimal intakes for selected micronutrients (i.e., thiamine, magnesium, iron, calcium, iodine, and selenium).

These findings are of clinical importance. For example, a man developed acute heart failure (cardiac beriberi) and abnormal neurological dysfunction (Wernicke’s encephalopathy) after cutting starchy foods from his meals for 3 months, with a 34 kg weight loss [11]. Every 0.1 mmol/l decrease in serum magnesium level was associated with an 18% increase in the likelihood of T2D (HR 1.18 [95% CI 1.04, 1.33]) and 12% for the likelihood of prediabetes (HR 1.12 [95% CI 1.01, 1.25]), with magnesium involved in the cellular mechanism of insulin secretion and insulin sensitivity [13, 29].

The half-life of thiamine is short (1–12 h), and the body can store it for 1 to 3 weeks, mostly in the heart, liver, muscle, and brain tissues [30, 31]. Thus, a regular dietary intake is needed to maintain optimal levels. Though thiamine can be found in meat, poultry, or eggs, it can be destroyed by the cooking process of high temperature and pH [31]. Tannin in tea and coffee can also inhibit the absorption of thiamine [31]. All of these factors could explain the relatively low thiamine concentration in LCD-followers. Of interest, those who followed a LCD in our study did not have an increased meat intake in their diet as a replacement for carbohydrate foods, instead adopting a hypocaloric approach associated with cutting or reducing carbohydrates.

Our data did not find evidence that participants who followed LCDs for longer periods had a lower thiamine status. Since thiamine has a short half-life and short time for body storage, following LCDs for at least 1 month might be enough to lead to a low thiamine status. Once thiamine intake is constantly low, regardless of duration, the thiamine status could set to a new equilibrium at a low level.

Low intakes of thiamine and magnesium among LCD-followers aligned with our previous systematic review of LCDs and micronutrient intakes, showing thiamine and magnesium intakes of 75–90% of the RNI [7]. Calcium, iron, and iodine intakes among LCD followers were also below RNI, similar to findings from the systematic review. The present study also showed that supplementation in LCD-followers improved micronutrient intakes towards meeting the RNI, but this was not true for all vitamins and minerals. With supplementation, the proportion of LCD-followers who met RNI increased from 57 to 69% for thiamine, from 39 to 57% for magnesium, and from 16 to 29% for iron intake, while the proportion meeting RNI for calcium and iodine remained unchanged. Similar to LCD-followers, the control group also had calcium (41%) and iodine intakes (33%) below the RNI and at a similar level to the LCD group (33% below RNI for calcium and 41% for iodine).

We did not measure plasma magnesium before and after (self-directed) magnesium supplement, so we cannot determine whether participants supplementing magnesium had a poorer pre-study magnesium supply than non-supplementers.

It is interesting to note that 7 participants in the LCD group and 6 in the control group were misclassified when they were re-allocated according to their actual carbohydrate intake being < 130 or ≥ 130 g/day. About 10% of people thus wrongly believe they are following LCDs or do not recognise that they are actually doing so. It is possible that there were errors in the dietary assessment methods, but these data more likely reflect poor knowledge of the basics of nutrition in the general population. Under 20% of self-declared LCD followers had discussed their diet choices with healthcare professionals. Most trusted information is from the internet or popular diet books. This is of particular concern if LCD followers could be at risk of micronutrient insufficiencies without properly informed guidance, and opens a debate about how people should be best guided over dietary choices within health services [17].

The following limitations should be considered when appraising the findings from this study, which aimed to describe population characteristics, dietary intake, and micronutrient status rather than seeking a causal relationship. The study recruited participants through local advertising calling for volunteers who were following or not following LCDs. This would inevitably attract individuals with a high interest in health, so the results might not apply to all people who adopt LCDs for other reasons. Dietary intake was self-reported, as is always the case for free-living people in real-life settings. This may introduce errors and bias towards underreporting by people with overweight or obesity, but body weight was similar in the two groups, and self-reporting is less likely otherwise to bias estimates of dietary thiamine consumption. The data apply to the UK and may not be translated to other regions, limiting external validity to other regions or countries with differences in cultures, beliefs about foods, food provision, and dietary assessment methods. Despite the limited generalisability of the dietary data, our findings proved, for the first time, that risks of thiamine and magnesium deficiencies do exist in individuals following LCDs, particularly in those not using supplementation. We recognise that in populations with greater seasonal variation in food consumption, it might be important to control for this. However, we recruited participants throughout the year, so the data broadly incorporate any seasonal fluctuations. Furthermore, there is very little variability in food consumption by season in the population studied. There could be other factors, such as alcohol and cigarette use, hormone replacement, bariatric surgery, physical activity, and other metabolic disorders, that might affect micronutrient status, besides dietary intake. These possible factors would add variance around the measures of micronutrient status. These interesting considerations cannot be addressed by the data available in the present study.

The present study also has several strengths. It proves the existence of relatively low blood biomarkers of thiamine status, with evidence of deficiency, in self-reported individuals following LCDs, alongside several vitamin and mineral intakes. Although LCDs can be effective for weight loss, health risks could differ from other weight loss diets, as discussed earlier. Our findings suggest a need to rethink the recommendation of LCDs in clinical practice. However, more research in real-life settings of LCD-followers is needed to extend and confirm these important findings. Furthermore, high-quality audits of practice with different diets in real-life settings could give a more complete picture of the safety and efficacy of dietary approaches for weight and diabetes management.

## Conclusion

There is evidence of relatively low thiamine intake and blood TDP level in self-reported individuals who restricted carbohydrate intake compared to those with normal carbohydrate intake. Biochemical thiamine deficiency was also documented in LCDs; however, this was not observed in individuals who took micronutrient supplements. These findings raise awareness of the importance of micronutrient sufficiency as one factor to consider before opting to follow LCDs.

## Electronic supplementary material

Below is the link to the electronic supplementary material.

Supplementary Material 1
