# Supplementary material for: Low thiamine status in adults following low-carbohydrate / ketogenic diets: a cross-sectional comparative study of micronutrient intake and status
Source: Eur J Nutr. 2024 Jul 5;63(7):2667–79. doi: 10.1007/s00394-024-03459-y (PMC11490449; doi:10.1007/s00394-024-03459-y)
Supplement: Supplementary file 1 — Supplementary Material 1 [file 394_2024_3459_MOESM1_ESM.docx]

**Supporting Information**

**Table S1. Post-hoc analysis of consumption of food groups in ‘true LCD’ followers (<130g CHO/day) and controls (n=94)**

| **Food groups as grams/day** | **True LCD-followers**  **n=45** | **Controls**  **n=49** | **P-value ^a^** |
| --- | --- | --- | --- |
|  | **<130 g CHO** | **≥130 g CHO** |  |
| Cereals and cereal products | 18 (0, 111) | 300 (219, 453) | ***<0.001*** |
| Potatoes | 9 (0, 18) | 26.4 (18, 63) | ***<0.001*** |
| Meat and meat products | 127 (79, 185) | 102 (58, 184) | 0.552 |
| Fish and fish products | 45 (24, 74) | 32 (24, 57) | 0.458 |
| Eggs and egg dishes. | 40 (22, 50) | 40 (18,50) | 0.488 |
| Fats and oils | 6 (4, 10) | 9 (4, 12) | 0.366 |
| Nuts and seeds | 14 (2, 28) | 12 (2, 24) | 0.553 |
| Vegetables | 315 (194, 446) | 255 (157, 405) | 0.309 |
| Fruits | 83 (47, 175) | 220 (113, 318) | ***<0.001*** |
| Soups and sauces | 33 (20,61) | 57 (32,128) | ***0.004*** |
| Milk and milk products | 74 (32, 122) | 77 (31, 160) | 0.672 |
| Non-alcoholic beverages | 675 (327, 880) | 359 (219, 653) | ***0.024*** |
| Sugars preserves and snacks | 6 (1, 11) | 27 (17, 51) | ***<0.001*** |

Data are median and inter quartile range generated from FFQ.

^a^ P-value of Mann-Whitney U test

**Table S2. Post-hoc analysis of macronutrient contribution in ‘true LCD’ followers (<130g CHO/day) and controls (n=98)**

| **Nutrients** | **Median of intakes per day** | | | **Proportion meeting recommendation,**  **n (%)** | | | **Recommendation** |
| --- | --- | --- | --- | --- | --- | --- | --- |
|  | **True LCD-followers**  **n=48** | **Controls**  **n=50** | **P-value ^a^** | **True LCD-followers**  **n=48** | **Controls**  **n=50** | **P-value** |  |
| Energy (kcal) | 1229 (987, 1423) | 1827 (1495, 2168) | ***<0.001*** | - | - | - | - |
| CHO (%E) | 15.8 (10.2, 28.9) | 49.6 (43.9, 53.1) | ***<0.001*** | 2 (4) | 23 (46) | <0.001 ^c^ | 50%E |
| Protein (%E) | 24.7 (21.4, 28.4) | 18.2 (15.6, 21.9) | ***<0.001*** | - | - | - | - |
| Fat (%E) | 55.3 (46.7, 63.2) | 33.7 (31.0, 39.0) | ***<0.001*** | 4 (8) | 31 (62) | <0.001 ^c^ | <35%E |
| SFA (%E) | 18.2 (13.5, 25.8) | 11.2 (9.4, 13.3) | ***<0.001*** | 5 (10) | 22 (44) | <0.001 ^d^ | <11%E |
| MUFA (%E) | 21.5 (18.6, 23.2) | 12.9 (11.8, 15.5) | ***<0.001*** | 44 (92) | 24 (48) | <0.001 ^d^ | 13% |
| PUFA (%E) | 8.2 (7.0, 10.3) | 6.5 (5.7, 7.5) | ***<0.001*** | 38 (79) | 25 (50) | 0.003 ^d^ | 6.5% |
| Fibre (g) ^b^ | 12.5 (8.5, 20.4) | 20.1 (16.1, 28.0) | ***<0.001*** | 2 (4) | 10 (20) | 0.028 ^c^ | 30 g/day |
|  |  |  |  |  |  |  |  |
| CHO (g) | 51.8 (27.9, 100) | 208 (162, 267) | ***<0.001*** | - | - | - | - |
| Protein (g) | 75.1 (58.7, 98.6) | 85.8 (67.8, 115) | 0.091 | - | - | - | - |
| Fat (g) | 76.4 (55.2, 91.5) | 66.2 (52.6, 93.7) | 0.469 | - | - | - | - |
| SFA (g) | 25.8 (16.1, 37.1) | 21.2 (15.9, 30.3) | 0.233 | - | - | - | - |
| MUFA (g) | 29.1 (23.9, 34.0) | 25.6 (18.9, 37.4) | 0.434 | - | - | - | - |
| PUFA (g) | 11.6 (8.2, 15.3) | 12.9 (11.4, 16.6) | 0.092 | - | - | - | - |

Data are median and inter quartile range. CHO, carbohydrate; E, energy; SFA, saturated fatty acids; MUFA, mono-unsaturated fatty acids; PUFA, poly-unsaturated fatty acids.

^a^ P-value of Mann-Whitney U test

^b^ Fibre is presented as AOAC method by multiplying non-starch polysaccharides (Englyst methods obtained from FFQ) by 1.33.

^c^ Fisher’s exact tests

^d^ Chi-square tests

**Table S3. Post-hoc analysis of intake of vitamins and minerals from diet in ‘re-allocated true’ LCD-followers (<130g CHO/day) and controls (n=98)**

|  | **True LCD-followers** | **Controls** | **p-value ^a^** |
| --- | --- | --- | --- |
|  | **n=48** | **n=50** |  |
|  | **<130g CHO** | **≥130 g CHO** |  |
| **Vitamins** |  |  |  |
| - A (mcg) ^b^ | 1455 (800, 1854) | 1029 (731, 1431) | 0.083 |
| - D (mg) | 3.5 (1.9, 5.5) | 2.7 (1.4, 4.0) | 0.087 |
| - B1 (mg) | 0.81 (0.65, 1.22) | 1.50 (1.19, 1.76) | ***<0.001*** |
| - Folate (mcg) | 251 (147, 329) | 271 (213, 325) | 0.233 |
| - B12 (mcg) | 8.1 (3.6, 12.9) | 5.4 (3.2, 8.5) | 0.092 |
| - C (mg) | 87.6 (55.1, 128) | 115 (64.6, 152) | 0.078 |
| **Minerals** |  |  |  |
| - Calcium (mg) | 484 (401, 701) | 687 (552, 903) | ***0.002*** |
| - Magnesium (mg) | 211 (147, 280) | 292 (258, 357) | ***<0.001*** |
| - Iron (mg) | 8.7 (6.3, 10.8) | 10.9 (9.1, 12.8) | ***0.001*** |
| - Copper (mg) | 0.9 (0.7, 1.4) | 1.4 (1.1, 1.7) | ***<0.001*** |
| - Zinc (mg) | 8.3 (6.3, 10.3) | 9.6 (7.5, 12.0) | ***0.028*** |
| - Selenium (mcg) | 54.8 (35.2, 77.2) | 76.0 (50.1, 92.9) | ***0.008*** |
| - Iodine (mcg) | 107 (68.4, 154) | 126 (100, 178) | 0.077 |

Data are median and interquartile range, obtained from food records, not including dietary supplements.

^a^ P-value of Mann-Whitney U test,

^b^ vitamin A as retinol equivalent

**Table S4. Intake of vitamins and minerals from diet only in women (n=65) and men (n=33)**

|  | **LCD followers** | **Controls** | **p-value ^a^** | **RNI** |
| --- | --- | --- | --- | --- |
| **Men (n=33)** | **N=13** | **N=20** |  |  |
| **Vitamins** |  |  |  |  |
| - A (mcg) ^b^ | 1602 (927, 1837) | 802 (680, 1267) | ***0.027*** | 700 |
| - D (mg) | 4.7 (1.9, 5.8) | 3.2 (1.5, 6.1) | 0.478 | 10 |
| - E (mg) | 9.1 (5.2, 13.7) | 10.8 (8.7, 15.5) | 0.316 |  |
| - B1 (mg) | 0.9 (0.7, 1.4) | 1.4 (1.2, 1.6) | 0.128 | 1 |
| - B2 (mg) | 1.3 (1.1, 2.2) | 1.5 (1.2, 2.0) | 0.928 | 1.3 |
| - B3 (mg) | 24.0 (20.8, 32.0) | 24.8 (20.1, 28.1) | 0.957 | 16.5 |
| - B6 (mg) | 1.6 (1.2, 2.7) | 2.2 (1.7, 2.5) | 0.372 | 1.4 |
| - Folate (mcg) | 254 (176, 351) | 250 (197, 302) | 1.000 | 200 |
| - B12 (mcg) | 10.7 (6.8, 13.9) | 6.5 (3.5, 11.6) | 0.298 | 1.5 |
| - C (mg) | 92.4 (60.1, 120) | 78.8 (52.9, 124.2) | 0.813 | 40 |
| **Minerals** |  |  |  |  |
| - Sodium (mg) | 2315 (1762, 3037) | 2432 (2103, 3151) | 0.548 | 2400 |
| - Potassium (mg) | 2744 (1902, 3754) | 3141 (2679, 3640) | 0.413 | 3500 |
| - Calcium (mg) | 561 (425, 845) | 758 (538, 1026) | 0.181 | 700 |
| - Magnesium (mg) | 255 (179, 329) | 302 (263, 353) | 0.334 | 300 |
| - Iron (mg) | 9.9 (6.5, 12.7) | 10.8 (9.2, 12.7) | 0.413 | 8.7 |
| - Copper (mg) | 1.5 (0.8, 1.6) | 1.2 (1.0, 1.7) | 0.624 | 1.2 |
| - Zinc (mg) | 9.8 (7.6, 11.5) | 11.2 (8.2, 12.9) | 0.573 | 9.5 |
| - Selenium (mcg) | 66.8 (39.2, 94.3) | 79.7 (50.9, 95.5) | 0.548 | 75 |
| - Iodine (mcg) | 132 (74.8, 171) | 139 (98.5, 197) | 0.413 | 140 |
|  |  |  |  |  |
|  | **LCD followers** | **Controls** | **p-value** | **RNI** |
| **Women (n=65)** | **N=36** | **N=29** |  |  |
| **Vitamins** |  |  |  |  |
| - A (mcg) ^b^ | 1455 (940, 2103) | 1035 (620, 1400) | ***0.018*** | 600 |
| - D (mg) | 3.2 (1.9, 5.7) | 2.4 (1.8, 3.2) | ***0.027*** | 10 |
| - E (mg) | 9.9 (7.9, 12.2) | 9.3 (7.6, 11.8) | 0.732 |  |
| - B1 (mg) | 0.98 (0.68, 1.40) | 1.33 (0.87, 1.65) | ***0.045*** | 0.8 |
| - B2 (mg) | 1.5 (1.0, 1.9) | 1.2 (0.9, 1.5) | 0.116 | 1.1 |
| - B3 (mg) | 21.5 (16.1, 23.6) | 22.0 (16.4, 26.5) | 0.864 | 13.2 |
| - B6 (mg) | 1.6 (1.4, 2.1) | 1.9 (1.4, 2.3) | 0.616 | 1.2 |
| - Folate (mcg) | 268 (222, 342) | 262 (174, 321) | 0.262 | 200 |
| - B12 (mcg) | 8.1 (3.8, 12.9) | 4.3 (3.1, 6.8) | ***0.030*** | 1.5 |
| - C (mg) | 110 (65.7, 148) | 114 (54.2, 151) | 0.958 | 40 |
| **Minerals** |  |  |  |  |
| - Sodium (mg) | 1989 (1402, 2515) | 1947 (1521, 2702) | 0.833 | 2400 |
| - Potassium (mg) | 2629 (2057, 3200) | 2813 (2335, 3505) | 0.436 | 3500 |
| - Calcium (mg) | 607 (418, 714) | 598 (483,738) | 0.741 | 700 |
| - Magnesium (mg) | 235 (177, 302) | 272 (207, 336) | 0.196 | 270 |
| - Iron (mg) | 9.1 (7.1, 11.8) | 9.5 (7.5, 11.4) | 0.895 | 14.8 |
| - Copper (mg) | 1.0 (0.9, 1.4) | 1.3 (0.9, 1.7) | 0.110 | 1.2 |
| - Zinc (mg) | 8.2 (6.9, 10.3) | 8.8 (7.0, 10.4) | 0.916 | 7 |
| - Selenium (mcg) | 56.9 (38.2, 79.1) | 63.2 (46.9, 86.8) | 0.406 | 75 |
| - Iodine (mcg) | 114 (68.0, 169) | 118 (71.2, 132) | 0.645 | 140 |

Data are presented as median and interquartile, obtained from food records, not including dietary supplements.

^a^ P-value of Mann-Whitney U test

^b^ vitamin A as retinol equivalent

**Table S5. Post-hoc analysis of blood concentration of thiamine, magnesium, zinc, copper, and selenium in true LCD-followers (<130g CHO/day) and controls (n=98)**

|  | **True LCD-followers**  **n=48** | **Controls**  **n=50** | **P-value** |
| --- | --- | --- | --- |
| RBC Thiamine (ng/g Hb) ^a^ | 399 (354, 472) | 566 (440, 729) | ***<0.001*** |
| Plasma magnesium (mmol/l) ^b^ | 0.80 (0.06) | 0.81 (0.05) | 0.209 |
| Plasma zinc (µmol/l) ^b^ | 12.9 (1.9) | 12.5 (1.5) | 0.307 |
| Plasma copper (µmol/l) ^a^ | 16.2 (13.4, 17.9) | 14.4 (12.4, 17.2) | 0.141 |
| Plasma selenium (µmol/l) ^a^ | 1.3 (1.2, 1.4) | 1.2 (1.1, 1.3) | 0.191 |

RBC, red blood cells.

The reference interval for thiamine is 275-675 ng/g Hb; for plasma Mg is 0.75-1.0 mmol/l; for plasma zinc is 11-18 µmol/l in men and 10-18 µmol/l in women; for plasma copper is 10-22 µmol/l in men and 11-25 µmol/l in women; for plasma selenium is 0.75–1.50 µmol/l.

^a^ Values are median (interquartile range) with p-value from Mann-Whitney U test

^b^ Values are mean (SD) with p-value from Independent T-test
